# Rapid Fragment Screening by ^19^F Steady‐State Free Precession NMR

**DOI:** 10.1002/anie.6891540

**Published:** 2026-05-26

**Authors:** Laura Ruduša, Sintija Ozola, Kostiantyn P. Melnykov, Yaroslav I. Filatov, Serhiy V. Ryabukhin, Raitis Bobrovs, Dmytro M. Volochnyuk, Kristaps Jaudzems, Rihards Aleksis

**Affiliations:** ^1^ Latvian Institute of Organic Synthesis Riga Latvia; ^2^ Faculty of Natural Sciences and Technology Riga Technical University Riga Latvia; ^3^ Enamine Ltd Kyiv Ukraine; ^4^ Taras Shevchenko National University of Kyiv Kyiv Ukraine; ^5^ Institute of Organic Chemistry National Academy of Sciences of Ukraine Kyiv Ukraine; ^6^ Faculty of Medicine and Life Sciences University of Latvia Riga Latvia

**Keywords:** drug discovery, 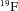
 NMR, fragment screening, SSFP

## Abstract

Steady‐state free precession (SSFP) NMR is emerging as a powerful tool across multiple fields of magnetic resonance, driven by its high sensitivity and advances in acquisition and processing. By continuously detecting steady‐state transverse magnetization, SSFP achieves superior signal‐to‐noise ratios per square‐root unit time compared to conventional NMR methods that require magnetization recovery between scans. Here, we demonstrate the application of 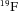 SSFP for fragment‐based screening, achieving broadband excitation (>120 kHz) and a 2.6–3.3‐fold average sensitivity enhancement relative to broadband Carr–Purcell–Meiboom–Gill approaches. This gain translates into up to a tenfold reduction in experimental time, enabling reliable screening of up to 2000 compounds per day. Together, these features establish 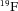 SSFP as a robust approach for rapid fragment screening in drug discovery.

## Graphical abstract

SSFP NMR is a broadband and very sensitive experiment for detecting protein–ligand interactions. The method enables rapid fragment screening at low micromolar protein concentrations, even for challenging drug targets such as intrinsically disordered proteins.

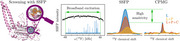

## 1 Introduction

In recent decades, fragment‐based drug discovery (FBDD) has become a key strategy for identifying lead compounds in drug development [1, 2, 3]. In comparison to traditional high‐throughput screening, FBDD provides higher hit rates and facilitates the exploration of a broader chemical space using smaller compound libraries. Furthermore, this approach has proven valuable for targeting challenging and typically undruggable proteins [4]. Among the available screening methods, nuclear magnetic resonance (NMR) stands out as one of the leading techniques for fragment‐based screening. A wide range of NMR experiments has been developed, broadly classified as protein‐observed or ligand‐observed methods. The former approach provides more detailed information on the protein–ligand interactions; however, it suffers from lower throughput and higher costs due to the need for target protein labeling. Therefore, ligand‐observed methods are typically preferred for faster and more cost‐effective NMR screening [5]. Among these, experiments exploiting the nuclear Overhauser effect (e.g., STD [6] and waterLOGSY [7, 8]) and the spin‐lattice relaxation in the rotating frame ($T_{1\rho }$) [9] are widely used 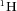 NMR screening methods. More recently, PEARLScreen [10] was introduced, which detects binding through changes in transverse relaxation time constants ($T_{2}$), enabling significantly higher binding contrast and lower protein concentrations.

**FIGURE 1:**
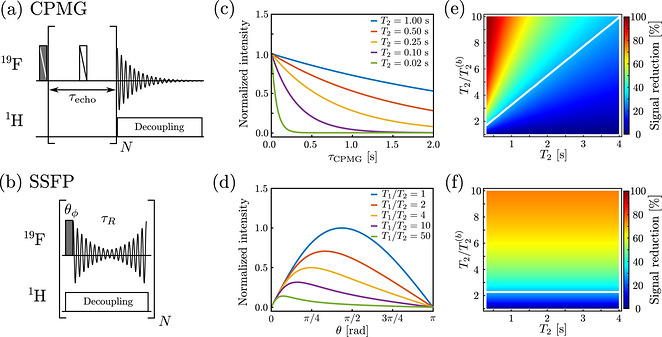
CPMG and SSFP pulses sequences and simulations. Pulse sequences for CPMG (a) and SSFP (b). The filled rectangle, filled rectangle with diagonal, empty rectangle with a diagonal represent conventional pulse, shaped excitation pulse and shaped refocusing pulse, respectively. In WURST‐CPMG the excitation pulse is conventional and refocusing pulses are WURST pulses, while in BURBOP‐CPMG excitation and refocusing pulses are shaped BURBOP pulses. θ represents the pulse flip angle and ϕ the phase, which in general is incremented. $\tau_{\text{echo}}$, $\tau_{R}$, and N are the echo delay in the CPMG sequence, the SSFP repetition time, and the number of times the sequence elements in brackets are repeated, respectively. The signal intensity dependence on the relaxation delay in CPMG (c) and the flip angle in SSFP (d) for different $T_{2}$ values but the same $T_{1}=1$ s. Relative signal reduction in CPMG (e) and SSFP (f) as a function of $T_{2}$ and the ratio $T_{2}/T_{2}^{\left(b\right)}$, where $T_{2}$ is the transverse relaxation time of a spin in a free molecule and $T_{2}^{\left(b\right)}$ is that for a molecule undergoing exchange between free and complex form with a biomolecule. The white line marks 30% signal reduction, which is our experimental cutoff for binding. The analytical simulations used a CPMG relaxation delay of 160 ms, and π/3 pulse flip angle for SSFP.

Protein and ligand consumption can be further reduced, and screening throughput increased, by monitoring binding via 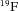 transverse relaxation [11] with the Carr–Purcell–Meiboom–Gill (CPMG) experiment (Figure 1a) [12, 13]. In the bound state, the 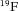 transverse relaxation rate is strongly enhanced due to the large chemical shift anisotropy of 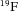. In addition, the substantial chemical shift differences between free and bound states lead to pronounced exchange contributions to the observed $T_{2}$. Thus, 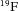 CPMG provides higher binding sensitivity than 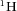 approaches [5], enabling shorter experimental times and lower ligand and protein concentrations. Moreover, the simplicity of 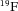 spectra, typically a single peak for each fragment, coupled with the large 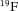 chemical shift dispersion (>300 ppm), allows screening mixtures with a higher number of compounds, 16–40 molecules per sample [14], compared with the 4–16 fragments in 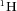 NMR experiments [10, 15]. However, conventional radiofrequency (RF) pulses often lack the bandwidth to uniformly excite the broad 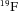 spectral range due to experimentally limited RF power. To address this, frequency‐swept pulses, such as wideband, uniform rate, and smooth truncation (WURST) [16] pulses, have been implemented in the CPMG block [17, 18]. Nevertheless, at high magnetic fields (>600 MHz NMR instruments), only about half of the required excitation bandwidth can be achieved. More recently, broadband universal rotations by optimal control (BURBOP) [19] pulses have been integrated into the CPMG sequence for both excitation and refocusing, providing uniform signal intensities over a bandwidth of 120 kHz, thereby covering the relevant 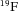 chemical shift range [20].

Despite these advancements, NMR screening throughput remains limited by its inherently low sensitivity. Several hyperpolarization strategies have been applied to fragment screening that exploit either parahydrogen or unpaired electrons as sources of high spin polarization. Signal amplification by reversible exchange (SABRE) [21] method provides more than a 1000‐fold enhancement through para‐hydrogen‐induced polarization, while dissolution dynamic nuclear polarization (DNP) [22, 23], achieves similar gains by transferring polarization from unpaired electrons. However, both techniques require specialized instrumentation and are incompatible with commercial autosamplers [24]. Moreover, dissolution DNP involves long polarization build‐up times (tens of minutes to hours), which negates throughput gains for typical protein and ligand concentrations. Photochemically induced DNP (Photo‐CIDNP) circumvents these limitations, and has achieved the highest screening throughput to date; however, the approach is restricted to specific molecular systems, narrowing the accessible chemical space [25].

Recently, steady‐state free precession (SSFP) [26] has re‐emerged as an alternative approach for improving sensitivity, with reported signal‐to‐noise ratio (SNR) enhancements of up to an order of magnitude [27]. SSFP employs a train of equidistant pulses separated by a repetition time (Figure 1b). Once the steady‐state magnetization is established, signals can be detected rapidly and continuously without long recovery delays, as required in conventional NMR. This leads to an enhanced SNR per square‐root unit time (${\mathrm{SNR}}_{t}$) compared to traditional acquisition methods. However, SSFP has inherent challenges, including truncation artifacts and low resolution due to short acquisition times, and strong offset dependence, which creates periodic “dark bands” in the spectrum where the signal vanishes. Previous studies have addressed these limitations by developing novel acquisition and processing strategies that provide improved spectral quality [28, 29, 30, 31]. Hence, SSFP has become a valuable tool in magnetic resonance imaging for medical diagnostics [32, 33], in solid‐state NMR for studying quadrupolar nuclei [34, 35] and high‐resolution liquid‐state NMR [30, 36]. While 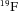 SSFP has been applied to qualitative [27] and quantitative [37] analysis of fluorinated compounds in environmental samples, as well as to MRI for tumor detection [38, 39] and in vivo cell tracking [40, 41], it has not, to date, been employed for fragment screening or for monitoring protein–ligand interactions.

In the present study, we demonstrate that 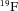 SSFP NMR enables protein–ligand binding studies with superior signal sensitivity than the current state‐of‐the‐art 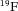 NMR methods. Our findings are supported by analytical theory and spin dynamic simulations. Furthermore, we assess the robustness and range of applications of 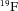 SSFP by screening a fluorinated compound library against seryl‐tRNA synthetase, an important antimicrobial drug target [42, 43] as well as microtubule‐associated protein Tau, an intrinsically disordered protein implicated in the progression of neurodegenerative diseases [44].

## 2 Results and Discussion

### 2.1 SSFP Theory and Applicability to Ligand Binding Detection

We begin by briefly reviewing the theory of the SSFP experiment and explore its application to 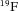 ligand‐observed monitoring of binding interactions. The SSFP sequence consists of a continuous train of pulses with flip angle θ and phase ϕ, spaced by a repetition time $\tau_{R}$ during which the signal is acquired (Figure 1b). The periodically applied pulses drive the build‐up of the steady‐state magnetization, which can reach half of the equilibrium value when the longitudinal $T_{1}$ and transverse $T_{2}$ relaxation time constants are equal [26]. Rapid and continuous detection of the steady‐state signal during the short repetition times ($\tau_{R}\ll T_{2}$) leads to superior ${\mathrm{SNR}}_{t}$ compared to conventional NMR acquisition. In addition, the use of short pulses (small flip angles, θ<π/2) enables broadband excitation of the 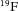 resonances. Both of these features are vital for fast 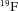 screening and are evaluated experimentally in the following section.

While SSFP can achieve broadband excitation, the periodic nature of the sequence produces “dark bands” in the frequency response, leading to regions of low or zero signal intensity. To achieve uniform excitation across the entire spectral range, we implement the multi‐acquisition SSFP approach [28, 29, 36, 45]. In this strategy, K separate SSFP experiments are recorded, each with a phase increment of $\Delta \phi_{n}=2\pi \left(n-1\right)/K$ in the $n^{\text{th}}$ experiment. The linear phase increment shifts the periodic frequency response by a constant offset, thus summing these K datasets averages out the strong offset dependence. We defer the interested reader to the Supporting Information and previous studies [28, 29, 45, 46] for more details. Throughout this study, we employ the SSFP variant with K=4, which ensures reasonably uniform signal intensities and phase (see Figures S1 and S4). Previous reports have also referred to this SSFP approach as Quadriga spectroscopy [28, 45].

In general, the SSFP signal intensity depends not only on the resonance offset but also on the pulse flip angle θ and spin relaxation properties, specifically, the relaxation time constant ratio ($T_{1}/T_{2}$) as described by

(1) $$S_{n}^{\prime }=\frac{\left(\exp(-i\left(\Phi -\Delta \phi_{n}\right))-1\right)\sin\theta }{\left(\cos(\Phi -\Delta \phi_{n})-1\right)\left(1+\cos\theta \right)+2\left(\cos\theta -1\right)\frac{T_{1}}{T_{2}}}.$$

Here, $S_{n}^{\prime }$ represents the signal intensity immediately after a pulse as defined in the Supporting Information, and Φ is the accrued phase. For simplicity, we have omitted the phase factor, which in practice is compensated by adjusting the receiver phase for each SSFP cycle. The average SSFP signal over K=4 experiments is given by

(2) $${\bar{S}}^{\prime }=\frac{1}{4}\sum_{n=1}^{4}S_{n}^{\prime }.$$

The maximum signal intensity in the SSFP experiment occurs when θ=π/2 and $T_{1}=T_{2}$. However, for $T_{1}/T_{2}$ values exceeding unity, the signal maximum decreases and is found at smaller flip angles, as shown in Figure 1d. The optimal flip angle for maximizing sensitivity can be calculated based on the $T_{1}$, $T_{2}$, and $\tau_{R}$ [47]. For low‐molecular‐weight compounds, such as those found in fragment libraries, 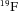 transverse relaxation times are typically long (>0.5 s) and comparable to longitudinal relaxation times, giving $T_{1}/T_{2}\approx 1$. Complex formation with a macromolecule slows ligand tumbling, markedly shortening $T_{2}$ and thereby increasing the $T_{1}/T_{2}$ ratio. In the CPMG experiment, this $T_{2}$ decrease causes a reduction in signal intensity, which is routinely exploited to monitor binding (Figure 1c). By analogy, in SSFP, the associated increase in $T_{1}/T_{2}$ attenuates the signal intensity, which can be leveraged to detect binding (Figure 1d). The potential signal reduction upon binding to the protein target is provided in Figure 1e,f, which shows 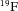 CPMG and SSFP signal dependence on the ligand 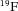 $T_{2}$ and on the $T_{2}/T_{2}^{\left(b\right)}$, where $T_{2}^{\left(b\right)}$ is the ligand transverse relaxation time in the presence of a protein. Binding is typically defined as a signal reduction greater than 30%, indicated by the white line in each plot. In the CPMG experiment, the reduction also depends on the relaxation delay $\tau_{\text{CPMG}}$, which here is set to 160 ms, within the standard 100–200 ms range used for binding detection. For short $T_{2}$ values (<0.8 s), CPMG has an advantage over SSFP in detecting binding for ligands that exhibit small $T_{2}$ changes ($T_{2}/T_{2}^{\left(b\right)}<2$), as is typical for weaker ligand–protein interactions. However, when $T_{2}>1.0$ s SSFP provides a significantly larger signal reduction and thus higher sensitivity to binding (Figure 1e,f).

### 2.2 Performance Evaluation of CPMG and SSFP Sequences

**FIGURE 2:**
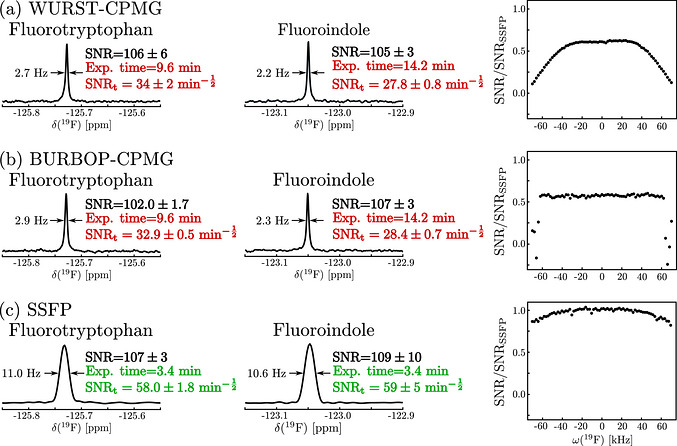
Performance comparison of different CPMG sequences and SSFP. For each pulse sequence from left to right are shown the 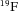 spectra of 5‐Fluoro‐L‐tryptophan and 6‐Fluoroindole and a plot with the maximum signal peak intensity as a function of the offset. For each 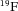 spectrum, the SNR, experimental time, ${\mathrm{SNR}}_{t}$ and linewidth are reported. In the panels on the right each experiment was acquired with the same experimental time and the signal intensities are normalized with respect to the SNR of the on‐resonance SSFP signal.

Next, we assess the sensitivity gains of SSFP compared to WURST‐ and BURBOP‐CPMG methods using 5‐Fluoro‐L‐tryptophan and 6‐Fluoroindole. The CPMG experiments were acquired with an inter‐experiment delay (acquisition time + recycle delay) of $1.3T_{1}$ to maximize the ${\mathrm{SNR}}_{t}$ [48]. As shown in Figure 2a,b, no significant differences are observed in the SNR between the two CPMG versions. Notice that the ${\mathrm{SNR}}_{t}$ of 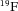 in fluorotryptophan is slightly higher than fluoroindole, which can be attributed to the threefold shorter 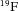 $T_{1}$ of the former molecule.

For SSFP experiments, maximum sensitivity is achieved when $\tau_{R}\ll T_{2}^{*}$, whereas the highest resolution is obtained for $\tau_{R}\gg T_{2}^{*}$ [45]. Here, $T_{2}^{*}$ denotes the total decay time constant, which includes inhomogeneous contributions. We observed that several fluorinated molecules exhibit 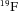 $T_{2}^{*}$ values in the range of 0.25 s. Hence, we selected $\tau_{R}=40$ ms as a compromise that provides good SNR for these compounds (see Figure S3), and sufficient resolution for analyzing fragment mixtures. Previous MRI studies have indicated that acquisition of the oscillatory SSFP signal from the initial transient regime may lead to spectral artifacts [49], therefore we discarded the first loops, corresponding to a duration on the order of 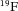 $T_{1}$. When compared to CPMG, SSFP demonstrates a notable improvement in ${\mathrm{SNR}}_{t}$, with enhancements of approximately 1.7‐fold for fluorotryptophan and 2.0‐fold for fluoroindole. Moreover, under identical experimental conditions the ${\mathrm{SNR}}_{t}$ values match between fluorotryptophan and fluoroindole, despite the distinct relaxation properties of 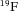. This is explained by the similar $T_{1}/T_{2}$ ratios in both systems, indicating that SSFP is more robust against variations in absolute relaxation parameters. Based on measurements from one of the compound mixtures, the $T_{2}$ values in our library range from 0.25 to 1.7 s, while the $T_{1}$/$T_{2}$ ratios are between 1 and 2. Consequently, an additional improvement in ${\mathrm{SNR}}_{t}$ over CPMG approaches could be attained when studying mixtures of fluorinated ligands, where optimal CPMG parameters cannot be used for each molecule. Nevertheless, a substantial increase in sensitivity is already observed with SSFP, leading to shorter experimental times by factors up to 4.

The use of short repetition times $\tau_{R}$ maximizes SSFP sensitivity, however, it also compromises spectral resolution. This is evident in Figure 2, where the signals in SSFP spectra exhibit broader linewidths than CPMG, increasing from 2.2–2.9 to 10.6–11.0 Hz. The inherently large 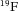 chemical shift dispersion (spanning over 100 kHz) ensures that the resolution of ca. 10 Hz is sufficient to prevent signal overlap for typical fragment mixture sizes containing 20–40 compounds per sample. In cases where resonance overlap becomes an issue, the SSFP resolution can be increased two‐ to threefold using longer $\tau_{R}$, with only a 10%–20% loss in sensitivity for compounds with the fastest signal decay rates (see Figure S3).

While the broad 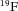 chemical shift range enables use of large fragment mixtures, it also poses challenges for exciting the entire spectrum using conventional pulses with practical power levels. Therefore, we continue by evaluating the excitation bandwidth of the SSFP sequence and broadband CPMG methods by acquiring 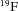 spectra of fluorotryptophan as a function of the offset. WURST‐CPMG [17, 18] sequence has an excitation bandwidth of approximately 80 kHz, within which 90% of on‐resonance signal intensity is retained (Figure 2a). As discussed previously [20], this bandwidth is insufficient to cover the entire pharmaceutically relevant 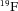 chemical shift range at high magnetic fields (>600 MHz NMR instruments). To address this, broadband pulses must also be used for the excitation pulse, as in BURBOP‐CPMG [20], which achieves uniform signal excitation across 120 kHz (Figure 2b). While SSFP employs conventional pulses with RF power of 20 kHz, the method achieves excitation of 90% of on‐resonance signal intensity over bandwidths of 120 kHz. This can be attributed to two factors: (i) the use of pulses with smaller flip angles and (ii) the splitting of the frequency response into periodic bands, which expands the excitation bandwidth, in analogy to the delays alternating with nutations for tailored excitation experiment [50, 51]. This is further supported by numerical spin dynamic simulations (Figure S4), which, as expected, demonstrate that shorter pulses (smaller flip angles) increase the excitation bandwidth. However, at smaller flip angles, the signal intensity and phase exhibit progressively larger periodic modulations. As shown in Figure 2c, in practice, pulses with flip angles ca. $60^{\circ }$ provide the necessary bandwidth on a 600 MHz NMR instrument without introducing unwanted phase distortions.

### 2.3 Protein–Ligand Binding Detection With CPMG and SSFP

**FIGURE 3:**
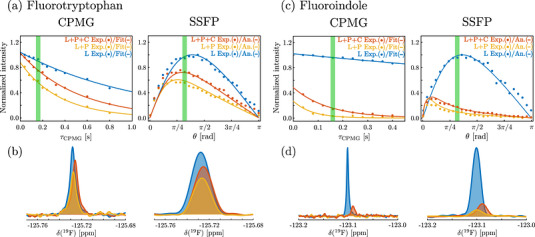
Binding detection with BURBOP‐CPMG and SSFP. (a) Experimental 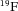 signal maxima of fluorotryptophan (L), fluorotryptophan with BSA (L+P) and fluorotryptophan with BSA and Naproxen (L+P+C) obtained with BURBOP‐CPMG and SSFP sequences. (c) Experimental 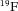 signal maxima of fluoroindole (L), fluoroindole with BSA (L+P) and fluoroindole with BSA and indole (L+P+C) obtained with BURBOP‐CPMG and SSFP sequences. The experimental signal amplitudes (filled circles) in (a,c) are plotted as a function of the $T_{2}$ relaxation delay $\tau_{\text{CPMG}}$ for BURBOP‐CPMG, and as a function of pulse flip angle θ for SSFP. The solid line in the CPMG data represents the best fit to a monoexponential decay, while in the SSFP data, it corresponds to the analytical model according to Equation (2), using the experimental $T_{1}$ and $T_{2}$ values. Spectra of fluorotryptophan (b) and fluoroindole (d) are shown for L (blue), L+P (yellow) and L+P+C (red) acquired with BURBOP‐CPMG using $\tau_{\text{CPMG}}=160$ ms, and with SSFP using $\theta \approx 60^{\circ }$. The values are highlighted in green in the panels in (a) and (c).

We have established that SSFP is sufficiently broadband to excite the relevant 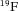 chemical shift range while also providing superior sensitivity to state‐of‐the‐art CPMG methods. Next, we examine its ability to monitor ligand binding to biological macromolecules. Indole and its derivatives, including tryptophan, exhibit strong binding affinity to bovine serum albumin (BSA), with $K_{D}$ values ranging from 20–200 μM [52, 53, 54], making this a commonly used model system for NMR‐based protein–ligand binding studies. Figure 3a shows the 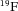 signal intensity of fluorotryptophan for BURBOP‐CPMG and SSFP as a function of the CPMG relaxation delay $\tau_{\text{CPMG}}$ and SSFP pulse flip angle θ, respectively. Three different cases are considered: (i) fluorotryptophan without BSA (marked as L), (ii) ligand with BSA (L+P), (iii) ligand with BSA and with a competitor compound (L+P+C). For BURBOP‐CPMG, within the typical $\tau_{\text{CPMG}}=100$–200 ms range [20] used for monitoring binding, the 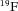 signal amplitude decreases to about 60% upon addition of BSA, while the signal recovers to approximately 80% in the presence of a high‐affinity competitor (Naproxen) [55]. Similar intensity changes are observed for SSFP with flip angles $45^{\circ }$–$65^{\circ }$, which also corresponds to the range that provides optimal sensitivity and excitation bandwidth. Representative spectra with $\tau_{\text{CPMG}}=160$ ms and $\theta \approx 60^{\circ }$ values, showing a clear intensity contrast upon binding are provided in Figure 3b for each method.

6‐Fluoroindole appears to be a more potent binder to BSA, as its signal vanishes in the presence of the protein in the BURBOP‐CPMG experiment when $\tau_{\text{CPMG}}>100$ ms (Figure 3c,d). The signal remains undetectable even after the addition of Naproxen (data not shown). However, with a 20‐fold excess of indole, approximately 12% of the 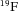 signal resurfaces. Similarly, a strong binding effect is observed in SSFP spectra acquired with pulse flip angles of $45^{\circ }$–$65^{\circ }$. The 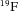 signal decreases to ca. 9% upon exposure to BSA, but recovers, doubling in amplitude after addition of a large excess of indole. Therefore, we conclude that both methods are equally effective in monitoring ligand binding.

Typically, an additional reference CPMG experiment is acquired for each sample using a short relaxation period (e.g., 20 ms). The analysis is then performed by evaluating the intensity ratio between experiments with short and long relaxation delays and comparing these ratios across samples. At the expense of increasing the screening time by a factor of two, this ratio‐of‐ratios approach effectively compensates for systematic artifacts, such as pipetting inaccuracies or poor shimming. In principle, analogous normalization schemes could be envisaged for SSFP by acquiring spectra with different flip angles; however, these relationships are more elaborate, particularly off‐resonance, which may complicate the analysis. Our results suggest that the contribution from systematic artifacts to the signal intensity remains below 10%, as indicated by the excellent agreement between experimental SSFP signal intensities and the analytical predictions, obtained from independently prepared samples of fluorotryptophan and fluoroindole (Figures 3 and S5). Additionally, in CPMG experiments, the integrated signal intensities agree when extrapolated to $\tau_{\text{CPMG}}$ = 0 (Figure S5) for all three samples in both cases. Together, these observations support that a single SSFP or CPMG experiment is sufficient for reliable protein–ligand binding detection, enabling a twofold increase in screening throughput.

### 2.4 Ligand Screening Against Folded Proteins: Seryl‐tRNA Synthetase

We next demonstrate the power of the SSFP experiment by screening a fluorinated fragment library against *E. coli* seryl‐tRNA synthetase (SerRS), which catalyzes the attachment of serine to its corresponding tRNA—a key step in protein synthesis [56]. Bacterial SerRS, along with other aminoacyl‐tRNA synthetases (aaRS), have been validated as viable antibacterial drug targets [42, 43]. Furthermore, the structural differences between the active sites of bacterial and eukaryotic aaRS [57] offer opportunities for the design of selective therapeutics.

**FIGURE 4:**
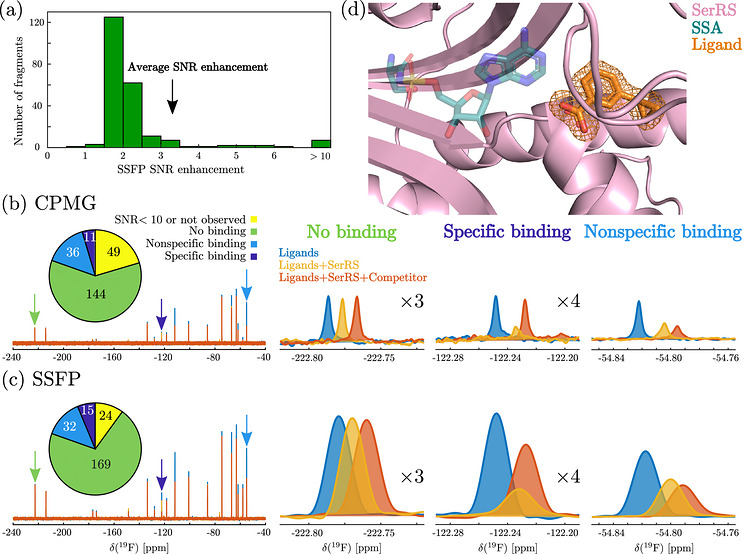
Fragment screening against seryl‐tRNA synthetase. (a) Histogram of SNR enhancement of SSFP over BURBOP‐CPMG. Pie charts showing the number of molecules exhibiting specific binding, nonspecific binding and no binding, or an SNR<10 (or not observed) for CPMG (b) and SSFP (c). Overlay of BURBOP‐CPMG (b) and SSFP (c) 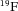 spectra for one representative fragment mixture recorded in the absence of SerRS (blue), in the presence of SerRS (yellow), and with both SerRS and SSA (red). Expanded spectral regions on the right illustrate examples of specific, nonspecific, and no binding. For clarity the spectra in yellow and red are shifted by 0.01 and 0.02 ppm relative to the blue spectrum. Intensities in the expanded spectral regions are scaled by a factor of C, as indicated by ×C. (d) Crystal structure of SerRS (pink) in complex with the identified hit (orange). SSA (green) from a previous structure (PDB ID: 6R1M) [58] is overlaid for comparison. The electron density corresponding to the hit ligand is shown as an orange mesh with $F_{o}-F_{c}$ polder omit electron density map depicted at 2σ level.

The fluorinated molecules employed in the NMR screening underwent thorough quality control (QC) to verify their structural integrity, purity, and solubility in DMSO and phosphate‐buffered saline. A total of 240 fragments that passed the QC were selected for the library. These molecules contain ${\mathrm{CF}}_{3}$, ${\mathrm{CF}}_{2}$, or CF groups, and mixtures of 20 compounds with different types of fluorine moieties were prepared under buffer conditions that ensure the stability of SerRS. However, the solution composition (30 mM Tris pH 9.0, 200 mM NaCl, 2 mM ${\mathrm{MgCl}}_{2}$) may not be optimal for some fragments. Therefore, we first assessed the SNR of the 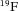 signal from each compound in the CPMG and SSFP spectra. To optimize sensitivity in CPMG, the 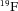 longitudinal relaxation time constants ($T_{1}$) were measured for all molecules in one of the samples, and the average relaxation time, ${\bar{T}}_{1}$, was calculated. The inter‐experiment delay in subsequent CPMG experiments was then set to $1.3{\bar{T}}_{1}$. We observed 16 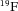 ligand signals in the SSFP spectra that were absent in the CPMG spectra. To obtain accurate SNR comparisons, separate longer experiments were recorded for these compounds. The histogram in Figure 4a summarizes the SNR enhancement observed with SSFP compared to CPMG for the fragment compound library. These data indicate that SSFP improves sensitivity on average by a factor of 3.3 when the run time of both experiments is equal. This translates to an average time saving of more than an order of magnitude. Finally, note that only a single molecule exhibited a lower SNR with SSFP as shown in the histogram in Figure 4a.

Since ligand binding is monitored through changes in signal intensity, we only considered compounds with a 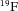 signal SNR above 10 to minimize false positive hits in our screening. A total of 216 out of 240 compounds met this criterion for SSFP, compared to 191 for CPMG. Fragments that did not fulfill this requirement were primarily those containing CF groups and exhibiting unfavorable relaxation parameters. For SSFP, 13% more compounds passed this second QC step, owing to its higher sensitivity and improved robustness to relaxation parameters. The experimental time could be increased to improve the SNR, thereby including more fragments in the screening analysis for both methods. However, we aimed for rapid screening, keeping the experiment time for each method below 4 min per sample, while using standard ligand concentrations of 50 μM.

For hit detection, an identical sample containing 5 μM SerRS was used. All samples were prepared from a common ligand cocktail stock solution, hence any systematic artifacts are expected to affect all peak intensities in a similar manner. To estimate this experimental variability, we evaluated the signal intensities of three compounds exhibiting the smallest changes between reference and protein‐containing samples across all mixtures. The resulting signal amplitude changes were below 7%, with average values of 1.8% and 2.4% for SSFP and CPMG, respectively. Fragment binding was defined as a signal amplitude decrease greater than 30% in the presence of the protein relative to the reference, a threshold that substantially exceeds experimental variability and thereby minimizes false positives. Binding was observed for 47 fragments (Figure 4b) with both methods. Generally, the hits detected by CPMG and SSFP overlapped, with SSFP identifying additional hits due to its higher SNR. However, several outliers were observed—fragments that showed binding in CPMG but not in SSFP—particularly among weaker binders with smaller signal intensity reductions. As discussed above, CPMG is more sensitive to binding detection for ligands with short $T_{2}$ values, which could explain this observation.

The development of new antibacterial agents requires identifying compounds that inhibit the catalytic activity of SerRS. Therefore, as a final step in our screening, we aimed to determine which hits bind to the active site of the synthetase. This was achieved by adding 5'‐O‐(N‐L‐seryl)‐ sulfamoyladenosine (SSA) [58], a known nanomolar inhibitor of *E. coli* SerRS, to the protein‐containing sample. We defined specific binding as an increase in signal intensity beyond the error margins of the signal amplitudes (95% confidence interval). In practice, this typically corresponded to identifying fragments with a signal recovery of 10% or more. As shown in Figure 4b, 15 and 11 specific hits were identified using SSFP and CPMG, respectively. The detected fragments matched well between the two methods, with only one CPMG hit not observed in SSFP. This fragment belonged to the class of weaker binders and would be classified as a specific binder in SSFP if the binding cutoff were lowered to 25%. Both methods exploit the same relaxation parameters in monitoring the ligand binding, and are therefore expected to identify identical hits. However, the enhanced sensitivity provided by SSFP within the same experimental time enabled the identification of over 36% more selective hits.

Finally, we sought to verify the binding specificity of the hits by determining the structure of ligand–SerRS complex with x‐ray diffraction. We employed the ligand‐soaking strategy, in which well‐diffracting protein crystals were incubated with the ligand solution, enabling the diffusion of the compound into the binding site through the solvent channels in the crystal [59]. Following this procedure the structure of one protein–ligand complex (Figure 4d) was obtained for an unfluorinated hit analogue (selected due to easier commercial availability). The ligand is positioned in the extension of the adenine‐binding subpocket as shown in Figure 4d. Although there is no direct steric overlap between SSA and the ligand (Figure 4d), SSA most likely blocks access to the binding cavity of the ligand, hence we observed competition in the NMR experiments. Notably, the pocket extension is absent in human SerRS, suggesting that this ligand and its derivatives could selectively inhibit bacterial SerRS, such as those from *E. coli* and *Staphylococcus aureus*, an essential prerequisite for the design of safe antibacterial agents with minimal side effects.

The total experimental time for the introduced SSFP screening protocol is approximately 20 min per fragment mixture, including NMR measurements of the three different samples (ca. 10 min), as well as sample exchange, tuning, and shimming on standard commercial instrumentation (ca. 10 min). This corresponds to a throughput of ca. 1400 molecules per day when screening mixtures of 20 fragments per sample to identify site‐specific binders, while the binding‐detection throughput exceeds 2000 compounds per day, which is the metric typically used to report screening throughput. We further streamlined the workflow by developing automated spectral processing and analysis in MATLAB [60], requiring only a few minutes for the entire dataset. Taken together, this enables a typical fragment library of 1000 compounds to be screened and analyzed within a single day. Potentially, the screening throughput could be further improved by a factor of approximately 2.7 through careful design of the fragment mixtures such that each molecule's 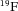 resonance coincides with the SSFP K=1 frequency response maxima (see Figure S1). However, this is difficult to realize in practice, as 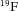 chemical shifts are very sensitive to experimental conditions such as pH, ionic strength, and temperature, which would reduce the robustness of the method and restrict the range of applicable screening conditions or compounds in the fragment library.

Previous efforts to address the inherently low sensitivity of NMR and improve screening throughput have focused on hyperpolarization strategies, including SABRE [21], dissolution DNP [61], and Photo‐CIDNP [25], which exploit either para‐hydrogen or unpaired electrons as polarization sources. To date, the most successful hyperpolarization‐based implementation for fragment screening has been Photo‐CIDNP [25], achieving a throughput of ca. 1500 molecules per day. This method, however, relies on recording ligand spectra from only two samples (with and without protein) and therefore identifies binders but not site‐specific binders. Under our instrumentation conditions (high magnetic field, cryogenically cooled probe), SSFP enabled a higher screening throughput than that reported for Photo‐CIDNP, with a binding‐detection throughput of over 2000 compounds per day. Furthermore, Photo‐CIDNP is restricted to a subset of molecules that can be photochemically polarized, thereby limiting the accessible chemical space. An analysis of the structural motifs present in approved drugs and compounds in clinical trials reveals that aliphatic and aromatic rings are represented in comparable proportions [62]. However, only about one third of aromatic fragments are Photo‐CIDNP active [25], implying that the method effectively probes less than 16% of the chemical space accessible by standard 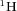 or 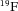 NMR screening approaches. Photo‐CIDNP does offer the clear advantage of enabling up to tenfold lower ligand concentrations than SSFP. However, the method requires higher protein concentration per ligand (2 μM/ligand in Photo‐CIDNP versus 0.25 μM/ligand in SSFP). In most screening campaigns, protein production costs typically outweigh those of ligands, thus the introduced 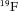 SSFP screening protocol is fast and cost‐effective.

### 2.5 Ligand Screening Against Intrinsically Disordered Proteins: Tau Protein

Approximately one third of the human proteome consists of intrinsically disordered proteins (IDPs)—highly dynamic biomolecules lacking stable secondary and tertiary structures [63]. Several IDPs have been implicated in cancer, diabetes, or neurodegenerative diseases [64]; however, their conformational plasticity makes them challenging drug targets, although small‐molecule binders are beginning to show promise [65]. Relaxation‐based NMR methods have emerged as powerful tools for identifying and characterizing small molecule binding to IDPs [66]. However, ligands remain extremely dynamic in the bound state, exhibiting fast rotational correlation times, such that changes in $T_{2}$ relaxation times are significantly smaller than those observed for folded proteins. As a result, higher ligand‐to‐protein concentration ratios are required to detect binding, substantially increasing protein consumption. We anticipate that the sensitivity gains afforded by 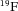 SSFP can alleviate this limitation by enabling the use of lower protein concentrations while maintaining fast and reliable screening. We assessed this potential by screening a fluorinated fragment library against the microtubule‐associated protein Tau, an IDP central to the pathology of Alzheimer's disease [44].

In this screening, we aimed to identify all fragments that bind to Tau, as the protein lacks a catalytic site, yet any small‐molecule binders may modulate its conformational landscape and reduce self‐aggregation. A high ligand‐to‐protein concentration ratio (1:1) was employed; however, to limit protein consumption, both Tau and ligand concentrations were set to 30 μM. Notably, the resulting protein concentration per ligand is comparable to that typically used in standard 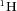 NMR screening methods for folded proteins. The experimental time for both CPMG and SSFP was increased to 6.5 min to compensate for the lower ligand concentrations, corresponding to a screening throughput of ca. 1500 compounds per day for each method, when including sample exchange, shimming, and tuning. In Tau screening, approximately 20% of fragment signals were absent or exhibited an SNR<10 in 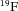 CPMG spectra, whereas only 7% fell below this threshold with SSFP. The average SSFP SNR enhancement over CPMG was 2.6 (Figure S6), once again underscoring the substantial sensitivity advantage.

**FIGURE 5:**
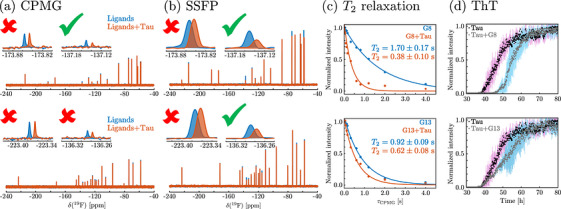
Fragment screening against Tau protein. CPMG (a) and SSFP (b) spectra of two representative fragment mixtures recorded in the absence (blue) and presence (red) of Tau. Insets show expanded spectral regions illustrating examples of no binding (left) and binding (right) as determined by SSFP. Red crosses and green ticks mark the no binding and hits, respectively. (c) Experimental 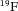 signal intensities (filled circles) for hits G8 (top) and G13 (bottom) plotted as a function of the CPMG relaxation delay $\tau_{\text{CPMG}}$ in the absence (blue) and presence (red) of Tau. Solid lines represent the best fit to a monoexponential decay. The extracted $T_{2}$ constants in absence (blue) and presence (red) are provided. (d) Thioflavin T fluorescence intensity as function of time in a solution of Tau alone and Tau in the presence of the hits G8 (top) and G13 (bottom). Filled circles mark the average normalized ThT fluorescence of triplicate data. The pink and blue area shows the error.

As in the SerRS screening, the experimental variability of the signal amplitudes between the two samples was small for all mixtures (below 2.4%), with the exception of a single sample for which the automatic shimming procedure performed poorly. In this case, the resulting spectral distortions were readily apparent, and the spectra were reacquired. Considering both the SerRS screening and a preliminary Tau screen (data not shown), shimming failures occurred in fewer than 1.5% of the samples, therefore occasional reacquisition does not significantly impact the overall screening throughput. Using the same binding criterion as before (≥30% signal reduction in presence of protein), we identified one hit using CPMG and two hits with SSFP, which we refer to as G8 and G13 (Figure 5a,b). The former was confirmed by both methods, however, G13 was not classified as a binder in CPMG as its 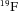 signal decreased by only 20%. To verify the SSFP hits, we measured their 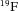 $T_{2}$ times in the presence and absence of Tau (Figure 5c). In both cases, the $T_{2}$ relaxation times decreased beyond the error margins, with G8 showing a particularly pronounced fourfold reduction in the $T_{2}$ value, indicating stronger binding affinity. Additionally, we acquired the 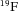 SSFP signal dependence on the pulse flip‐angle, which matched the analytical prediction from Equation (2) (Figure S7). These results confirm that the intensity changes observed in screening originate from faster relaxation.

We next evaluated whether the identified hits affect Tau aggregation. Thioflavin T (ThT) fluorescence was employed to monitor the aggregation of pseudophosphorylated Tau mutant [67]. In the presence of a fivefold excess of G8, the aggregation lag phase increased by approximately 7 h (Figure 5d), whereas G13 did not induce a significant change. These observations are consistent with the 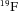 $T_{2}$ results and suggest that G8 exhibits a higher binding affinity toward Tau and can alter the conformational ensemble of the protein. Together, these results demonstrate that 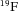 SSFP enables fast and reliable detection of fragment binders even for IDPs. More broadly, this methodology opens the door to routine NMR‐based screening of IDPs, a class of targets traditionally considered highly challenging.

## 3 Conclusion

Fragment‐based screening by NMR has long been limited by its inherently low sensitivity, restricting both throughput and scalability. In this work, we establish 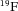 SSFP as a highly sensitive alternative to the widely used CPMG experiment, achieving an average SNR enhancement of 2.6–3.3‐fold, corresponding to average time savings of up to an order of magnitude. Moreover, SSFP employing RF pulses with only 20 kHz power provides sufficient excitation bandwidth for 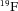 on a 600 MHz NMR instrument and is expected to remain sufficient even at higher magnetic fields. Incorporating frequency‐swept pulses into the SSFP sequence could further extend the excitation bandwidth while mitigating RF inhomogeneity effects, which have previously been shown to impact SSFP performance [68]. Leveraging its high sensitivity and the use of fragment mixtures (particularly when combined with cryogenically cooled probes), 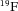 SSFP enables screening throughput approximately 30% higher than reported for state‐of‐the‐art hyperpolarization‐based methods such as Photo‐CIDNP [25]. While 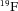 SSFP requires the presence of fluorine within the fragment structure, this does not substantially reduce the accessible chemical space. In contrast, Photo‐CIDNP is restricted to specific molecular structures, significantly limiting the chemical space.

One of the main limitations of SSFP is its reduced spectral resolution under conditions ($\tau_{R}\ll T_{2}^{*}$) that maximize sensitivity. Nevertheless, mixtures containing up to 20 compounds can be reliably screened, and even larger mixtures (30–40 fragments) may be feasible, particularly when longer repetition times $\tau_{R}$ are employed. Moreover, resolution can be improved by adopting alternative SSFP acquisition and processing strategies, such as phase‐incremented SSFP [30] or complete reduction to amplitude frequency table [27]. These approaches not only offer improved spectral resolution but may also further enhance sensitivity, making them promising avenues for future fragment‐based screening. A second limitation of the SSFP approach introduced here is the absence of explicit compensation for systematic artifacts, such as pipetting errors or suboptimal shimming. In practice, however, we did not observe significant artifacts arising from sample preparation, and shimming failures occurred in fewer than 1.5% of samples. These cases were readily identified and corrected by reacquisition, without a noticeable impact on overall screening throughput. Further improvements in shimming algorithms may enhance robustness and potentially eliminate shimming instabilities between samples altogether.

Despite these limitations, SSFP as implemented here stands at the forefront of fragment‐based drug discovery using NMR. We anticipate that SSFP will be increasingly employed for rapid and reliable 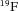 screening of conventionally druggable targets, while also paving the way for studying ligand–protein interactions in challenging systems such as IDPs.

## Conflicts of Interest

The authors declare no conflict of interest.

## Supporting information


**Supporting File**: Supporting information includes the analytical analysis of SSFP, sample preparation and all experimental details as well as additional simulated and experimental data. The authors have cited additional references within the Supporting Information [69, 70, 71, 72, 73, 74, 75, 76, 77, 78, 79, 80, 81, 82, 83, 84].

## Data Availability

The data supporting the findings of this study are available from the corresponding author upon reasonable request. The atomic coordinates and structure factors are deposited in the Protein Data Bank with accession code 30XK.
