# Supplementary material for: Rapid Fragment Screening by 19F Steady‐State Free Precession NMR
Source: Angew Chem Int Ed Engl. 2026 May 26;65(30):e6891540. doi: 10.1002/anie.6891540 (PMC13383281; doi:10.1002/anie.6891540)
Supplement: Supplementary file 1 — Supporting File: Supporting information includes the analytical analysis of SSFP, sample preparation and all experimental details as well as additional simulated and experimental data. The authors have cited additional references within the Supporting Information [69, 70, 71, 72, 73, 74, 75, 76, 77, 78, 79, 80, 81, 82, 83, 84]. [file ANIE-65-e6891540-s001.pdf]

Supporting information of “Rapid fragment screening by ^{19}F steady-state free precession NMR.”

Laura Ruduša,[1,2] Sintija Ozola,[1] Kostiantyn P. Melnykov,[3,4] Yaroslav I. Filatov,[3,5] Serhiy V. Ryabukhin,[3,4,5] Raitis Bobrovs,[1] Dmytro M. Volochnyuk,[3,4,5] Kristaps Jaudzems,[1,6] and Rihards Aleksis[1,a)]

[1)] *Latvian Institute of Organic Synthesis, Aizkraukles 21, Riga LV-1006, Latvia*

[2)] *Faculty of Natural Sciences and Technology, Riga Technical University, Paula Valdena 3, Riga LV-1048, Latvia*

[3)] *Enamine Ltd, 78 Winston Churchill street, 02094 Kyiv, Ukraine*

[4)] *Taras Shevchenko National University of Kyiv, 60 Volodymyrska street, 01033 Kyiv, Ukraine*

[5)] *Institute of Organic Chemistry, National Academy of Sciences of Ukraine, 5 Academik Kukhar street, 02094 Kyiv, Ukraine*

[6)] *Faculty of Medicine and Life Sciences, University of Latvia, Jelgavas 1, Riga LV-1004 Latvia*

(Dated: April 19, 2026)

[a)] Electronic mail: aleksis@osi.lv

I. THEORETICAL BACKGROUND OF SSFP.

A. Analysis of the SSFP sequence with constant pulse phase

Here, we revisit the theoretical framework of the SSFP experiment, closely following previous analyses[1,2]. Generally, the SSFP pulse sequence consists of a train of pulses with flip angle θ and phase ϕ, and are separated by the repetition time τ_{R}. Initially, we consider the case when all pulse phases are identical and equal to zero ($\phi = 0$).

We assume that after M SSFP steps, a steady-state has been established. At the beginning of the next step $(M+1)$, immediately before the $(M+1)^{\mathrm{th}}$ pulse, the density operator is given by:

$$\hat{\rho}_0 = a_z^{(0)}\hat{I}_z + a_x^{(0)}\hat{I}_x + a_y^{(0)}\hat{I}_y. \tag{S1}$$

Here $\hat{I}_l$ represents nuclear spin angular momentum operator along axis l. The identity operator is omitted, as it commutes with all other terms. After the $(M+1)^{\mathrm{th}}$ pulse, the density operator $\hat{\rho}_1$ has Cartesian components proportional to the coefficients $\left\{a_z^{(1)}, a_x^{(1)}, a_y^{(1)}\right\}$, which are expressed as:

$$a_z^{(1)} = a_z^{(0)}\cos\theta + a_y^{(0)}\sin\theta, \tag{S2}$$

$$a_x^{(1)} = a_x^{(0)}, \tag{S3}$$

$$a_y^{(1)} = a_y^{(0)}\cos\theta - a_z^{(0)}\sin\theta, \tag{S4}$$

After free evolution period τ_{R} of the $(M+1)^{\mathrm{th}}$ step, the operator coefficients of the density operator $\hat{\rho}_2$ are given by:

$$a_z^{(2)} = 1 - E_1 + a_z^{(1)}E_1, \tag{S5}$$

$$a_x^{(2)} = E_2\left(a_x^{(1)}\cos\Phi - a_y^{(1)}\sin\Phi\right), \tag{S6}$$

$$a_y^{(2)} = E_2\left(a_x^{(1)}\sin\Phi + a_y^{(1)}\cos\Phi\right). \tag{S7}$$

Here Φ is the accumulated phase, given by $\Phi = \tau_{\mathrm{R}}\Omega_{\mathrm{S}}$, where Ω_{S} is the resonance frequency. Relaxation effects are included phenomenologically by $E_1 = \exp(-\tau_{\mathrm{R}}/T_1)$ and $E_2 = \exp(-\tau_{\mathrm{R}}/T_2)$, where T_1 and T_2 are longitudinal and transverse relaxation time constants, respectively. Since there exists a steady-state and the Cartesian operators are orthogonal, each operator amplitude must be equal in the density operator at the beginning

($\hat{\rho}_0$) and at the end ($\hat{\rho}_2$) of the $(M+1)^{\text{th}}$ step. We are interested in describing the density operator after the $(M+1)^{\text{th}}$ pulse ($\hat{\rho}_1$), hence we substitute the coefficients $a_e^{(0)}$ with $a_e^{(2)}$ in Eqs. S2-S4, which gives a system of linear equations:

$$\begin{cases} a_z^{(1)} = a_z^{(2)} \cos\theta + a_y^{(2)} \sin\theta \\ a_x^{(1)} = a_x^{(2)} \\ a_y^{(1)} = a_y^{(2)} \cos\theta - a_z^{(2)} \sin\theta \end{cases} \tag{S8}$$

Finally, substituting the operator amplitudes $a_e^{(2)}$ with Eqs.S5-S7 and simplifying, we obtain a system of linear equations, which is given in matrix form by:

$$\begin{pmatrix} E_1\cos\theta - 1 & E_2 \sin\theta \sin\Phi & E_2 \cos\Phi \sin\theta \\ 0 & E_2\cos\Phi - 1 & -E_2 \sin\Phi \\ -E_1 \sin\theta & E_2 \cos\theta \sin\Phi & E_2 \cos\theta\cos\Phi - 1 \end{pmatrix} \begin{pmatrix} a_z^{(1)} \\ a_x^{(1)} \\ a_y^{(1)} \end{pmatrix} = \begin{pmatrix} (1-E_1)\cos\theta \\ 0 \\ (E_1 - 1)\sin\theta \end{pmatrix} \tag{S9}$$

Solving the system of linear equations yields the steady-state density operator $\hat{\rho}_1$, with the corresponding Cartesian product operator amplitudes given by:

$$a_z^{(1)} = \frac{(1-E_1)\left(\cos\theta\left(1 - E_2\cos\Phi\right) + E_2\left(E_2 - \cos\Phi\right)\right)}{1 - E_1\cos\theta - (E_1 - \cos\theta)\, E_2^2 + (E_1 - 1)\, E_2\, (1+\cos\theta)\cos\Phi}, \tag{S10}$$

$$a_x^{(1)} = \frac{(1-E_1)\, E_2 \sin\theta \sin\Phi}{1 - E_1\cos\theta - (E_1 - \cos\theta)\, E_2^2 + (E_1 - 1)\, E_2\, (1+\cos\theta)\cos\Phi}, \tag{S11}$$

$$a_y^{(1)} = \frac{(1-E_1)\sin\theta\, (E_2\cos\Phi - 1)}{1 - E_1\cos\theta - (E_1 - \cos\theta)\, E_2^2 + (E_1 - 1)\, E_2\, (1+\cos\theta)\cos\Phi}. \tag{S12}$$

In practice the NMR signal is acquired by using quadrature detection, which combines the two transverse components as follows:

$$M_+ = M_x + iM_y, \tag{S13}$$

where M_x and M_y are transverse magnetization components and in SSFP will be proportional to $a_x^{(1)}$ and $a_y^{(1)}$, respectively. Therefore, the detected SSFP signal at the beginning of each evolution period ($t = 0$) is given by:

$$S(0) = \frac{-\exp\left(i\pi/2\right)(E_1 - 1)\left(E_2 \exp(-i\Phi) - 1\right)\sin\theta}{1 - E_1\cos\theta - (E_1 - \cos\theta)\, E_2^2 + (E_1 - 1)\, E_2\, (1+\cos\theta)\cos\Phi}. \tag{S14}$$

Now, the signal at any time t during the evolution period is given by:

$$S(t) = S(0) \exp\left(i\Omega_{\text{S}} t\right) \exp\left(-t/T_2\right) \tag{S15}$$

This describes the SSFP signal when the pulse phase is constant throughout the experiment.

B. Analysis of the SSFP sequence with linearly incremented pulse phase

Next, we consider an SSFP experiment in which the pulse phase is incremented linearly. For the kth pulse the phase is given by:

$$\phi_k = \frac{2\pi(k-1)}{K}, \tag{S16}$$

where K is the number of SSFP steps until a full cycle is completed (i.e., $k = 1, \ldots, K$). Since each pulse is separated by the repetition time τ_{R}, then the time dependence of the pulse phase can be described as:

$$\phi(t) = \frac{2\pi t}{\tau_{\text{R}} K}. \tag{S17}$$

For simplicity, we have assumed pulses are ideal (infinitely short) and the kth pulse is located at time $t = (k-1)\tau_{\text{R}}$. In the rotating-frame the Hamiltonian for a pulse at time t is given by:

$$\hat{H}_1 = \theta \hat{R}_z(\phi(t)) \hat{I}_x \hat{R}_z(\phi(t))^{-1}. \tag{S18}$$

Each pulse will induce a rotation about the x-axis through an angle θ (or an axis in the xy-plane related to x via ϕ). The evolution during the repetition periods is governed by:

$$\hat{H}_0 = \Omega_{\text{S}} \hat{I}_z. \tag{S19}$$

As discussed previously[3], the description of the spin dynamics throughout the SSFP experiment can be simplified by transforming the rotating-frame Hamiltonian into the frequency-modulated frame:

$$\hat{H}^{(\text{fm})} = \hat{R}_z(\phi(t))^{-1} \hat{H}^{(\text{rot})} \hat{R}_z(\phi(t)) - \dot{\phi}(t) \hat{I}_z. \tag{S20}$$

In the frequency-modulated frame, the Hamiltonians that describe the spin dynamics throughout the repetition period and due to the pulses are given by:

$$\hat{H}_0^{(\text{fm})} = \left(\Omega_{\text{S}} - \omega_{\text{rf}}\right) \hat{I}_z, \tag{S21}$$

$$\hat{H}_1^{(\text{fm})} = \theta \hat{I}_x, \tag{S22}$$

where ω_{rf} is the transmitter offset due to the pulse phase dependence and is defined as $\omega_{\text{rf}} = \dot{\phi}(t) = \frac{2\pi}{\tau_R K}$. Although an additional offset term has emerged, all pulses in this frame

share the same phase. Thus, we can apply the same analysis as in Section I A to derive the density operators and SSFP signal equations. If we first transform the density operator in Eq.S1 into the frequency-modulated frame at time $t = 0$, then following the steps outlined in Section I A, the Cartesian product operator amplitudes of the density operator at a time immediately after a pulse are given by:

$$a_z^{(1)(\mathrm{fm})} = \frac{(1-E_1)\left(\cos\theta\left(1-E_2\cos\left(\Phi-\Delta\phi\right)\right)+E_2\left(E_2-\cos\left(\Phi-\Delta\phi\right)\right)\right)}{1-E_1\cos\theta-\left(E_1-\cos\theta\right)E_2^2+\left(E_1-1\right)E_2\left(1+\cos\theta\right)\cos\left(\Phi-\Delta\phi\right)}, \tag{S23}$$

$$a_x^{(1)(\mathrm{fm})} = \frac{(1-E_1)\,E_2\sin\theta\sin\left(\Phi-\Delta\phi\right)}{1-E_1\cos\theta-\left(E_1-\cos\theta\right)E_2^2+\left(E_1-1\right)E_2\left(1+\cos\theta\right)\cos\left(\Phi-\Delta\phi\right)}, \tag{S24}$$

$$a_y^{(1)(\mathrm{fm})} = \frac{(1-E_1)\sin\theta\left(E_2\cos\left(\Phi-\Delta\phi\right)-1\right)}{1-E_1\cos\theta-\left(E_1-\cos\theta\right)E_2^2+\left(E_1-1\right)E_2\left(1+\cos\theta\right)\cos\left(\Phi-\Delta\phi\right)}, \tag{S25}$$

where $\Delta\phi$ is the pulse phase increment ($\Delta\phi = \tau_R\omega_{\mathrm{rf}} = \frac{2\pi}{K}$). Next we transform the density operator out of the frequency-modulated frame at time $t = (k-1)\tau_R$ immediately after the kth pulse:

$$\hat{\rho}_1^{(k)} = \hat{R}_z(\phi((k-1)\tau_R))\hat{\rho}_1^{(\mathrm{fm})}\hat{R}_z(\phi((k-1)\tau_R))^{-1}, \tag{S26}$$

and thus the Cartesian product operator amplitudes are given by:

$$a_z^{(1)(k)} = \frac{(1-E_1)\left(\cos\theta\left(1-E_2\cos\left(\Phi-\Delta\phi\right)\right)+E_2\left(E_2-\cos\left(\Phi-\Delta\phi\right)\right)\right)}{1-E_1\cos\theta-\left(E_1-\cos\theta\right)E_2^2+\left(E_1-1\right)E_2\left(1+\cos\theta\right)\cos\left(\Phi-\Delta\phi\right)}, \tag{S27}$$

$$a_x^{(1)(k)} = \frac{(1-E_1)\,E_2\sin\theta\left(E_2\sin\left(\Phi-\Delta\phi-\frac{2\pi(k-1)}{K}\right)+\sin\frac{2\pi(k-1)}{K}\right)}{1-E_1\cos\theta-\left(E_1-\cos\theta\right)E_2^2+\left(E_1-1\right)E_2\left(1+\cos\theta\right)\cos\left(\Phi-\Delta\phi\right)}, \tag{S28}$$

$$a_y^{(1)(k)} = \frac{(1-E_1)\sin\theta\left(E_2\cos\left(\Phi-\Delta\phi-\frac{2\pi(k-1)}{K}\right)-\cos\frac{2\pi(k-1)}{K}\right)}{1-E_1\cos\theta-\left(E_1-\cos\theta\right)E_2^2+\left(E_1-1\right)E_2\left(1+\cos\theta\right)\cos\left(\Phi-\Delta\phi\right)}, \tag{S29}$$

As before, from this we derive the detected SSFP signal at the beginning of each evolution period $t = (k-1)\tau_R$ is given by:

$$S_k = \frac{-\exp\left(i(\pi/2+\Delta\phi(k-1))\right)\left(E_1-1\right)\left(E_2\exp(-i(\Phi-\Delta\phi))-1\right)\sin\theta}{1-E_1\cos\theta-\left(E_1-\cos\theta\right)E_2^2+\left(E_1-1\right)E_2\left(1+\cos\theta\right)\cos(\Phi-\Delta\phi)}. \tag{S30}$$

The signal at any time throughout the SSFP experiment is then given by:

$$S\left((k-1)\tau_R+t\right) = S_k\exp\left(i\Omega_{\mathrm{S}}t\right)\exp\left(-t/T_2\right), \tag{S31}$$

Notice that due to the periodicity of the SSFP sequence, the frequency response becomes periodic, with the signal intensity completely vanishing at the minima (Fig.S1). In multi-acquisition SSFP[4–6], several experiments are recorded with different phase increments to

shift the frequency response. If K is large enough then the sum of these experiments leads to uniform excitation across the spectrum (Fig.S1). If a multi-acquisition SSFP variant with K phase increments and K separate SSFP experiments is selected, then we define the phase increment of the nth experiment as $\Delta\phi_n = 2\pi(n-1)/K$.

The signal expression can be simplified when $\tau_{\mathrm{R}} \ll T_2 \leq T_1$, a condition typically met for repetition times used in practice in liquid-state NMR. Under this assumption, the exponential functions can be expanded as a Taylor series and truncated to first order. Hence, the SSFP signal at time $t = 0$ is expressed as:

$$S'_k(0) = \frac{-\exp\left(i(\pi/2 + \Delta\phi_n(k-1))\right)\left(\exp(-i(\Phi - \Delta\phi_n)) - 1\right)\sin\theta}{\left(\cos(\Phi - \Delta\phi_n) - 1\right)(1 + \cos\theta) + 2\left(\cos\theta - 1\right)\frac{T_1}{T_2}} \tag{S32}$$

Eq.S32 highlights the explicit dependence of the SSFP signal on the T_1/T_2 ratio, which forms the basis for the sensitivity to ligand binding to biomacromolecules exploited in this work.

In practice, the phase of the signal is typically compensated via the receiver phase, and signals acquired with different phase increments are co-added. The resulting averaged SSFP signal at time $t = 0$ is given by:

$$\overline{S}'(0) = \frac{1}{K}\sum_{n=1}^{K} \frac{\left(\exp(-i(\Phi - \Delta\phi_n)) - 1\right)\sin\theta}{\left(\cos(\Phi - \Delta\phi_n) - 1\right)(1 + \cos\theta) + 2\left(\cos\theta - 1\right)\frac{T_1}{T_2}} \tag{S33}$$

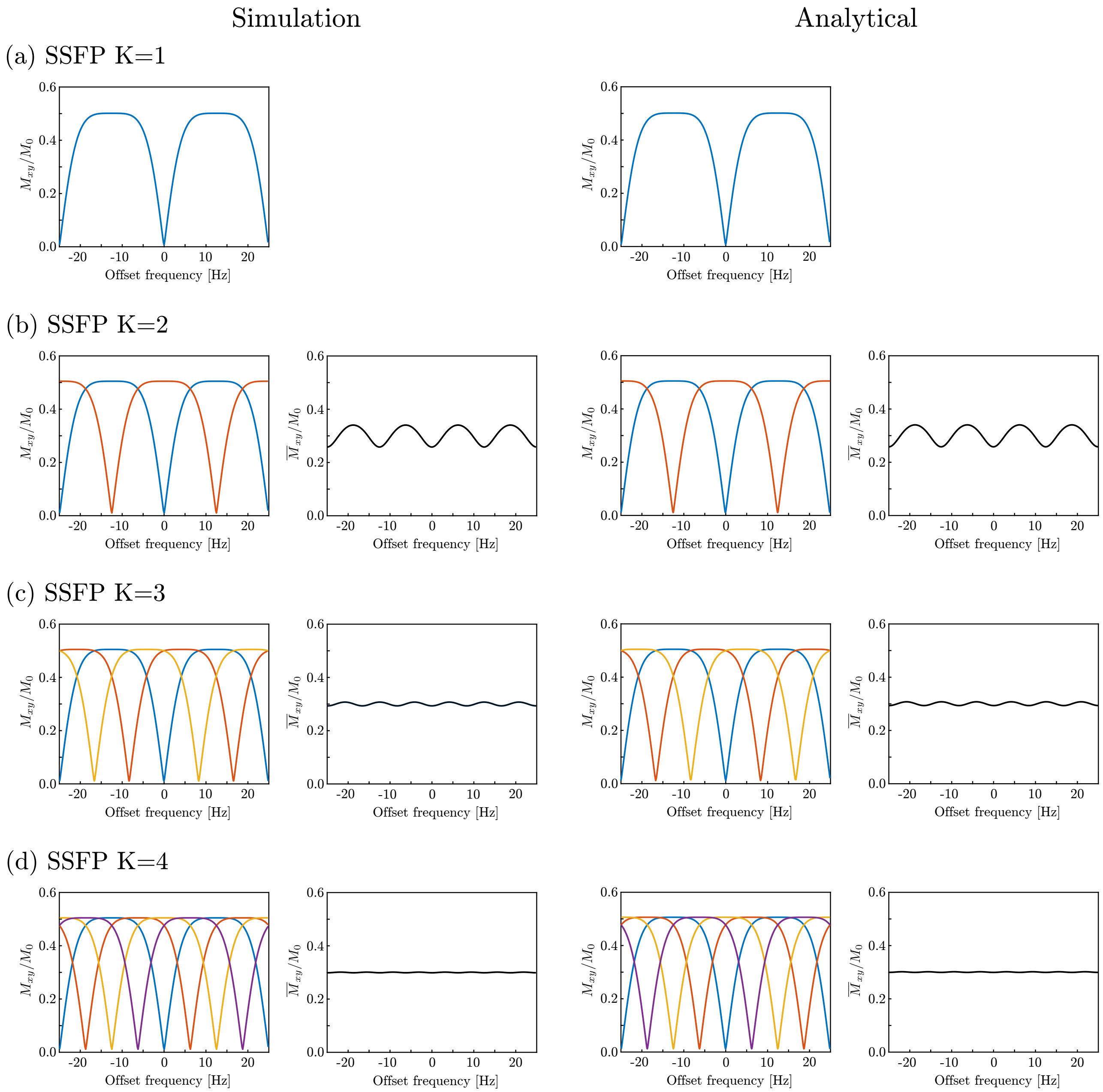

Figure S1. SSFP transverse magnetization as a function of resonance offset when $K = 1$ (a), $K = 2$ (b), $K = 3$ (c) and $K = 4$ (d). M_{xy}/M_0 is the transverse magnetization normalized to equilibrium magnetization. $\overline{M}_{xy}/M_0$ is normalized average transverse magnetization of K experiments. Different plot line colors correspond to different phase increments. Panel with plot in black on the right for simulation and analytical data shows the average frequency response of all experiments in each case. The simulations utilized $\tau_R = 40$ ms, $\theta = \frac{\pi}{2}$ and $T_1 = T_2 = 2$ s.

II. METHODS

E. coli SerRS expression and purification

E. coli SerRS with N-terminal 6xHis-tag was cloned in pET-3a vector and overexpressed in E. coli NiCo21(DE3) strain (New England Biolabs, USA). Cells were grown until OD_{600} reached 0.8, then cooled to 20°C and 0.1 mM IPTG was added. Expression was carried out for 16 h at 20°C, after which cells were harvested by centrifugation. Cell pellets were resuspended in buffer A (30 mM Tris pH 9.0, 200 mM NaCl, 30 mM imidazole, 2 mM $MgCl_2$) and lysed by sonication. Lysate was centrifuged at 30000 $\times$g for 30 min and supernatant was loaded on previously equilibrated HisTrap column (Cytiva). Protein was eluted with buffer B (30 mM Tris pH 9.0, 200 mM NaCl, 300 mM imidazole, 2 mM $MgCl_2$) gradient. Collected fractions were concentrated and applied to previously equilibrated HiLoad 16/600 Superdex 200 pg column using buffer C (30 mM Tris pH 9.0, 200 mM NaCl, 2 mM $MgCl_2$). For crystallization, 6xHis-tag was cleaved off with TEV protease (made in-house)[7] overnight at 4°C, using 1:50 SerRS/TEV molar ratio. Cleaved SerRS was purified using reverse HisTrap. Protein in the flow-through fraction was collected and concentrated to 6 mg/mL.

2N4R Tau and its mutant expression and purification

2N4R Tau and its PHF mutant (with mutations S396D, S400D, S404D)[8] used for aggregation was cloned in pET-17b vector and overexpressed in E. coli T7 Express strain (New England Biolabs, USA). Cells were grown until OD_{600} reached 1.8, then 0.1 mM IPTG was added. Expression was carried out at 37°C for 3 hours, after which cells were harvested by centrifugation. The protein was purified following a previous study[9] with slight modifications. Cell pellets were resuspended in lysis buffer (8 M urea, 50 mM NaPi pH 6.9, 1 mM EDTA, 0.1 mM PMSF, 5 mM DTT) and lysed by sonication for 20 min (2 s on, 4 s off). Lysate was centrifuged at 30000 $\times$g for 30 min, supernatant was dialyzed overnight against buffer A (50 mM NaPi pH 6.9, 1 mM EDTA, 0.1 mM PMSF, 5 mM DTT). The following day the dialyzed mixture was heated at 75°C for 15 min, cooled on ice and centrifuged at 30000 $\times$g for 30 min to remove insoluble impurities. Supernatant was then applied to previously equilibrated SP HP column. Protein was eluted with buffer B (50 mM NaPi pH 6.9, 1 M NaCl 1 mM EDTA, 5 mM DTT) gradient. Collected fractions were concentrated and ap-

plied to previously equilibrated HiLoad 26/600 Superdex 200 pg column using buffer C (1× PBS pH 7.4, 10 mM DTT). Protein-containing fractions were pooled and buffer exchanged to 1× PBS pH 7.4, 4 mM TCEP for NMR applications.

Quality control of fluorine compound library

The compound library was constructed from the Enamine "in-stock" collection (ca. 3.8 million compounds). Initially, all molecules containing at least one fluorine atom were selected (ca. 850 000 compounds, Set 1). A filtration step using modified REOS filters (116 SMARTS patterns) removed highly reactive or toxic compounds unsuitable for subsequent biochemical assays, yielding 767 064 compounds (Set 2)[10,11]. Next, custom filters (33 SMARTS patterns) were applied to exclude compounds with elevated reactivity (e.g., arylators), multi-peak fragments, and water-unstable species, reducing the library to 618 182 compounds (Set 3)[11]. An additional water-stability filter left 616 655 compounds (Set 4)[12]. In the final physicochemical filtering stage, threshold criteria were enforced: MW ≤ 300, cLogP ≤ 3, HBA ≤ 5, HBD ≤ 3, resulting in 90 669 compounds (Set 5). Fluorinated fragments were characterized by 57 SMARTS groups (52 observed) using RDKit for all filtering steps except physicochemical property calculations, which were performed with DataWarrior[13,14]. A diverse subset of 300 compounds (Set 6) was chosen using RDKit's MaxMinPicker algorithm, incorporating fluorinated SMARTS patterns. The workflow is summarized in Fig.S2.

Finally, quantum-chemical predictions of ^{19}F-NMR chemical shifts were carried out in ORCA[15] (geometry optimization at B3LYP/def2-SVP, CPCM + GIAO B3LYP/ma-def2-TZVP). Parallel experimental NMR screening confirmed sole appropriate signals for 276 of the 300 compounds (24 compounds failed QC due to residual $CF_3CO_2^-$ signals from HPLC purification with TFA as a co-eluent, as well as insufficient solubility and stability).

Based on predicted shifts, 14 20-compound "cocktails" were proposed; experimental validation supported 12 of them, corresponding to a success rate of ca. 85.7%.

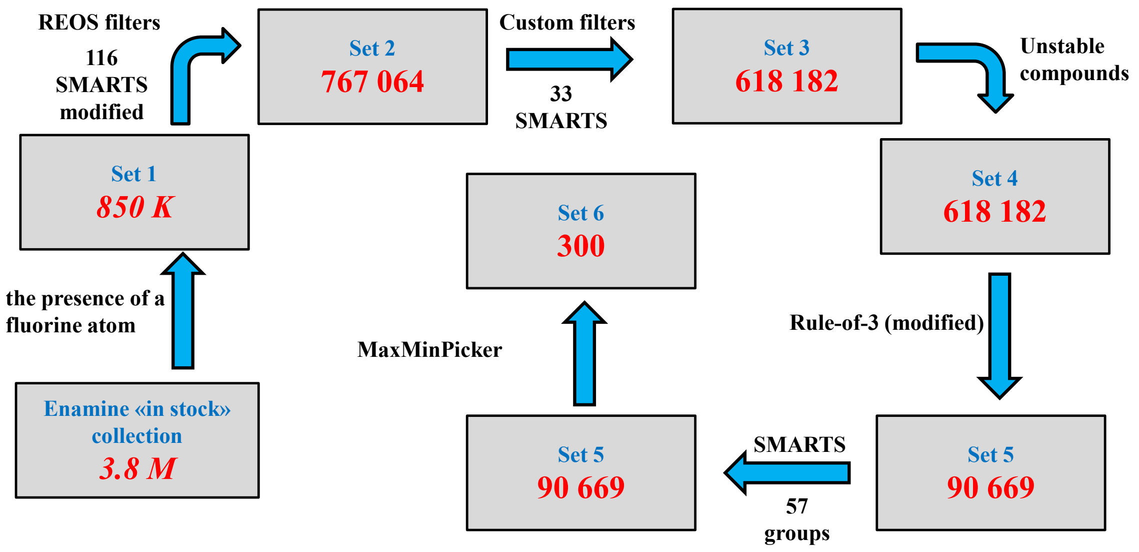

Figure S2. Multistage workflow of compound filtration and selection. The number of compounds remaining after each iteration is highlighted in red.

NMR spectroscopy

Samples for Bovine Serum Albumin (Sigma) screening tests were prepared in 3 mm NMR tubes and contained 5-Fluoro-L-tryptophan (Sigma, $>98\%$) or 6-Fluoroindole (Sigma, $>98\%$) at 100 μM concentration in 100 mM sodium phosphate pH 7.4 and 10% D_2O solution. Three identical samples were prepared for 5-Fluoro-L-tryptophan: (i) without BSA, (ii) with 5 μM BSA and (iii) with 5 μM BSA and 200 μM Naproxen (Sigma). Three identical samples were also prepared for 6-Fluoroindole: (i) without BSA, (ii) with 2.5 μM BSA and (iii) with 2.5 μM BSA and 2 mM Indole (Sigma).

Samples for SerRS screening were prepared in 3 mm NMR tubes and contained mixtures of 20 fragments dissolved at 50 μM concentration each in 30 mM Tris pH 9.0, 200 mM NaCl, 2 mM $MgCl_2$ and 10% D_2O solution. For each fragment cocktail two identical such samples were prepared without SerRS and in the presence of 5 μM SerRS. For the competition experiments, a solution of SSA in D_2O was directly added to the sample with SerRS to a final concentration of 100 μM.

Samples for 2N4R Tau screening were prepared in 3 mm NMR tubes and contained mixtures of 20 fragments dissolved at 30 μM concentration each in 1×PBS pH 7.4, 4 mM TCEP and 10% D_2O solution. For each fragment cocktail two identical such samples were

prepared without Tau and in the presence of 30 μM Tau.

NMR experiments were performed on 14.1 T magnet interfaced to a Bruker Avance Neo console equipped with a quadruple resonance CryoProbe (CP QCI 600S3 H/F-C/N-D-05 Z). The spectrometer operates at Larmor frequencies of 599.93 MHz and 564.50 MHz for ^{1}H and ^{19}F, respectively. The ^{19}F chemical shifts were referenced to ^{19}F signal of trifluoroacetic acid at -76.6 ppm. Unless stated otherwise all experiments utilized pulses with a radiofrequency (RF) amplitude of 20 kHz for ^{19}F. Furthermore, during ^{19}F acquisition in all experiments 4 kHz ^{1}H decoupling using garp4[16] was employed.

The WURST-CPMG experiments were carried out using 2 ms WURST-20 pulses sweeping through 150 kHz as refocusing pulses. BURBOP-CPMG experiments utilized BURBOP pulses for both excitation and refocusing as described previously[17]. Sensitivity evaluation experiments were conducted with 288 scans, 0.9 s recycle delay and acquisition time of 0.84 s for 5-Fluoro-L-tryptophan and 144 scans, 4.6 s recycle delay and acquisition time of 0.84 s for 6-Fluoroindole. 16 dummy scans were employed prior to acquisition. The echo delay τ_{echo} was 20 ms and 8 CPMG loops were used. SSFP experiments were performed with a repetition time of $\tau_R = \tau_{\text{acq}} + \tau_{\text{d}}$, where τ_{acq} is acquisition time set to 40 ms and τ_{d} is the dead time to mitigate probe ringing effects, which was set to 50 μs. Pulse flip angle θ was $\approx 60°$. 80 SSFP loops were used to establish the steady-state and 1200 were employed for acquisition. The ^{19}F T_1 and T_2 relaxation time constants were measured for each sample using inversion recovery and BURBOP-CPMG sequences, respectively. Inversion recovery experiments were acquired with 12 scans, 20 s recycle delay and 14 relaxation time points (0.05 to 20 s) for all samples. BURBOP-CPMG experiments used the same experimental parameters as stated above for each sample with 12 relaxation time points (0.04 to 2 s) for 5-Fluoro-L-tryptophan samples and 14 relaxation time points (0.04 to 4 s) for 6-Fluoroindole sample without BSA and 10 relaxation time points (0.04 to 0.4 s) for 6-Fluoroindole samples with BSA. Experiments for investigating the offset dependence were performed on 500 μM 5-Fluoro-L-tryptophan in 100 mM sodium phosphate pH 7.4 and 10% D_2O with identical experimental parameters as above except WURST-CPMG and BURBOP-CPMG used 40 scans, and SSFP employed 80 dummy loops and 400 acquisition loops. For both CPMG and SSFP the spectral window was 156250 Hz.

For SerRS screening BURBOP-CPMG experiments were performed with 96 scans and 0.9 s recycle delay. SSFP experiments were acquired with 120 dummy loops and 1200

acquisition loops, which were split over three separate acquisitions to avoid an issue with console memory. All other parameters were identical to experiments described above for BSA. The ^{19}F T_1 relaxation time constants of fragments in one of the mixtures were measured using saturation recovery sequence with 128 scans, 2 s recycle delay and 12 relaxation time points (0.001 to 20 s).

For Tau screening BURBOP-CPMG experiments were performed with 192 scans and 0.9 s recycle delay. All other parameters were identical to experiments described above. SSFP experiments were acquired with 80 dummy loops and 2400 acquisition loops, which were split over two separate acquisitions to avoid an issue with console memory. All other parameters were used as above. The ^{19}F relaxation time constants of the hits were determined on 50 μM ligand (G8 or G13) in the same buffer as used in the screening. T_1 constants were measured by the inversion recovery sequence with 96 scans, 20 s recycle delay and 10 relaxation time points (0.05 to 20 s). T_2 constants were measured with BURBOP-CPMG sequence with 400 scans, 0.9 s recycle delay and 10 relaxation time points (0.05 to 4 s).

All spectra were processed using in-house written MATLAB scripts. For CPMG experiments the FID was zero-filled and weighted by a decaying exponential function to maximize the SNR, subsequently Fourier transformed and phased. SSFP data were processed following the approach described previously[17] with slight modifications. First the FID was weighted by a decaying exponential function (3-4 Hz line broadening), subsequently forward linear prediction (8000-10000 points predicted) was employed using the matrix pencil method as implemented in MatNMR[18]. Finally, the FID was weighted by a trapezoidal function with left limit of 0 and right limit 0.35 and then Fourier transformed and phased. First-order phase correction is needed as a result of the dead time and signal phase dependence on the offset in SSFP.

The SNR of the ^{19}F peaks was calculated as follows:

$$\mathrm{SNR} = \frac{\max\left(\mathrm{real}\left(\mathrm{signal}\right)\right)}{\sigma\left(\mathrm{real}\left(\mathrm{noise}\right)\right)} \tag{S34}$$

where $\max\left(\mathrm{real}\left(\mathrm{signal}\right)\right)$ is the maximum peak intensity in the real part of the spectrum, $\sigma\left(\mathrm{real}\left(\mathrm{noise}\right)\right)$ is the standard deviation of the real part of the noise spectrum, which was estimated from a spectral range absent of any signals.

E. coli SerRS crystallization

SerRS crystals in apo form were grown in 0.1 M HEPES pH 7.5, 27.5% PEG Smear Medium (Molecular Dimensions, UK) using 6 mg/mL SerRS. For soaking, fragments were added in 20 mM final concentration directly in the well with the crystals. Crystals were soaked with the ligand for 30 min and then transferred to the cryoprotecting buffer well with 20% glycerol before freezing in liquid nitrogen. Diffraction data were collected at Diamond Light Source (Oxford, UK) beamline i03. To solve the protein structure, molecular replacement was carried out using the previously deposited SerRS structure (PDB ID: 6R1M)[19]. Phenix[20,21] and Coot[22] software were used for the refinement to improve the structure.

ThT fluorescence assay

PHF mutant of 2N4R tau (S396D, S400D, S404D) was used for thioflavin T (ThT) assay to promote aggregation, using an adapted protocol reported previously[23]. Each well in the assay contained 100 μM PHF, 25 mM HEPES (pH 7.2), 20 mM DTT, 25 μM ThT and 500 μM fragment (replaced by appropriate volume of DMSO for negative controls). Greiner 96-well microplates (F-bottom, chimney-well, black) were used, 100 μL of sample was added in each well together with a glass bead (diameter 4 mm, Sigma-Aldrich, USA) to promote aggregation. Measurements were carried out on Clariostar Plus plate reader (BMG Labtech, Germany) using excitation and emission wavelengths of 440 and 480 nm, respectively. Data points were recorded every 5 minutes for total duration of 80 hours, and measurements were carried out in triplicate.

Simulations

All numerical simulations were performed using SpinDynamica 3.9.0[24] in Mathematica 14.0. Relaxation in the simulations is included phenomenologically as implemented in Spindynamica. The relaxation time constants used in the simulations are stated in the corresponding figure captions.

III. ADDITIONAL SIMULATIONS AND EXPERIMENTAL DATA.

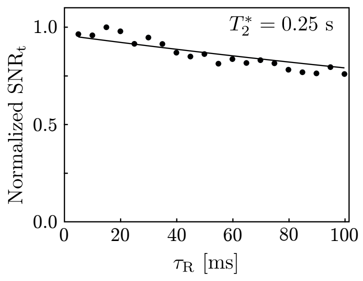

Figure S3. $\mathrm{SNR_t}$ dependence on the repetition time τ_R in SSFP. The black circles are normalized experimental ^{19}F $\mathrm{SNR_t}$ of 5-fluoro-L-tryptophan. Solid line represents analytically predicted $\mathrm{SNR_t}$ as a function of τ_R. In this model, steady-state transverse magnetization (M_ss) is assumed to be independent of τ_R and so $\mathrm{SNR_t} = \frac{M_\mathrm{ss} T_2^*}{\tau_\mathrm{R}}(1 - \exp(-\tau_\mathrm{R}/T_2^*))$. ^{19}F T_2^* was estimated from the signal linewidth processed without line broadening.

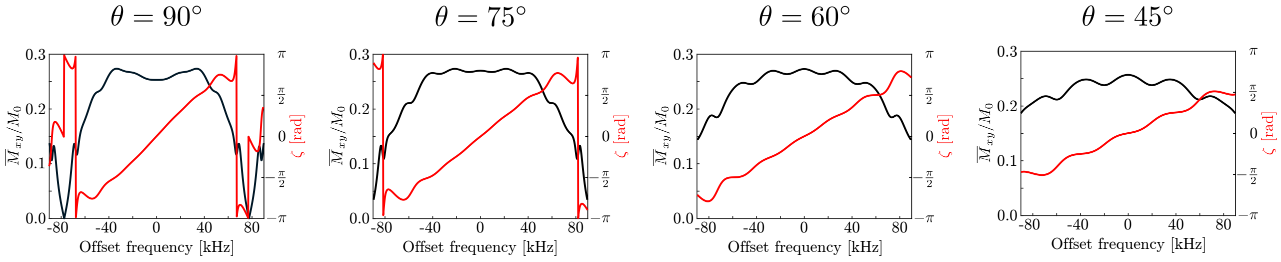

Figure S4. Numerical simulations of the frequency response in the SSFP experiment for different flip angles θ. The plots show the average normalized transverse magnetization amplitude ($\overline{M}_{xy}/M_0$) and its phase (ζ) as a function of the resonance offset. The simulations utilized $\tau_\mathrm{R} = 40$ ms, 20 kHz pulses with finite length and $T_1 = 1.42$ s, $T_2 = 1.06$ s, corresponding to experimental T_1 and T_2 values of ^{19}F in 5-Fluoro-L-tryptophan.

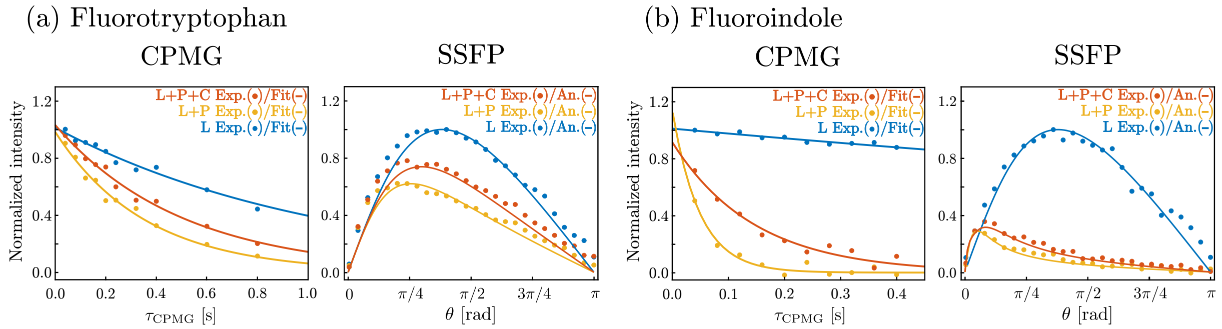

Figure S5. (a) Experimental ^{19}F signal integral of 5-fluoro-L-tryptophan (L), fluorotryptophan with BSA (L+P) and fluorotryptophan with BSA and Naproxen (L+P+C) obtained with BURBOP-CPMG and SSFP sequences. (c) Experimental ^{19}F signal integral of 6-fluoroindole (L), fluoroindole with BSA (L+P) and fluoroindole with BSA and indole (L+P+C) obtained with BURBOP-CPMG and SSFP sequences. The experimental signal integral (filled circles) in (a,c) are plotted as a function of the T_2 relaxation delay τ_{CPMG} for BURBOP-CPMG, and as a function of pulse flip angle θ for SSFP. The solid line in the CPMG data represents the best fit to a monoexponential decay, while in the SSFP data, it corresponds to the analytical model according to Eq.S33, using the experimental T_1 and T_2 values.

Table I. ^{19}F T_1 and T_2 relaxation constants in 5-Fluoro-L-Tryptophan and 6-fluoroindole.

Sample	Fluorotryptophan		Fluoroindole	
	T_1 [s]	T_2 [s]	T_1 [s]	T_2 [s]
Without BSA	1.42 ± 0.19	1.06 ± 0.12	4.5 ± 0.5	2.7 ± 0.2
With BSA	1.33 ± 0.16	0.36 ± 0.04	4.0 ± 0.5	0.049 ± 0.015
With BSA and Competitor	1.30 ± 0.14	0.50 ± 0.05	4.2 ± 0.5	0.15 ± 0.03

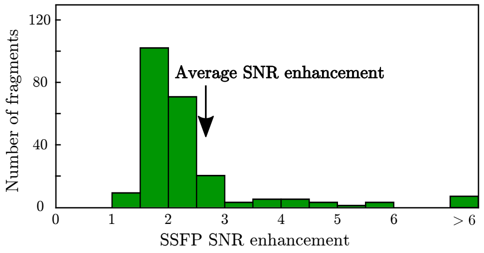

Figure S6. Histogram of SNR enhancement of SSFP over BURBOP-CPMG of ^{19}F signal of fragment from Tau screening.

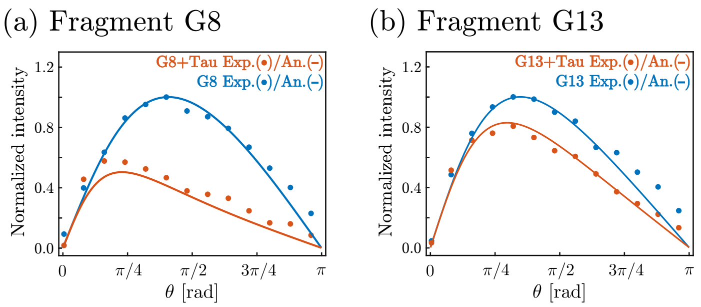

Figure S7. The experimental ^{19}F signal intensities (filled circles) of hits G8 (a) and G13 (b) are plotted as a function of pulse flip angle θ for SSFP. The solid line corresponds to the analytical model according to Eq.S33, using the experimentally determined T_1 and T_2 values given in Table II.

Table II. ^{19}F T_1 and T_2 relaxation constants of fragments G8 and G13.

Sample	G8		G13	
	T_1 [s]	T_2 [s]	T_1 [s]	T_2 [s]
Without Tau	2.03 ± 0.17	1.70 ± 0.17	1.66 ± 0.11	0.92 ± 0.09
With Tau	1.8 ± 0.2	0.38 ± 0.10	1.64 ± 0.16	0.62 ± 0.08

REFERENCES

[1]W. S. Hinshaw, "Image formation by nuclear magnetic resonance: The sensitive-point method," J. Appl. Phys. **47**, 3709–3721 (1976).

[2]A. Schwenk, "Steady-state techniques for low sensitivity and slowly relaxing nuclei," Prog. Nucl. Magn. Reson. Spectrosc. **17**, 69–140 (1985).

[3]Y. Zur, M. Wood, and L. Neuringer, "Motion-insensitive, steady-state free precession imaging," Magn. Reson. Med. **16**, 444–459 (1990).

[4]A. Schwenk, "NMR pulse technique with high sensitivity for slowly relaxing systems," J. Magn. Reson. **5**, 376–389 (1971).

[5]N. K. Bangerter, B. A. Hargreaves, S. S. Vasanawala, J. M. Pauly, G. E. Gold, and D. G. Nishimura, "Analysis of multiple-acquisition SSFP," Magn. Reson. Med. **51**, 1038–1047 (2004).

[6]T. B. Moraes, F. V. C. Kock, K. S. Salome, A. Barison, A. Simpson, and L. A. Colnago, "Steady-state free precession sequences for high and low field NMR spectroscopy in solution: Challenges and opportunities," J. Magn. Reson. Open **14**, 100090 (2023).

[7]J. E. Tropea, S. Cherry, and D. S. Waugh, "Expression and purification of soluble his6-tagged tev protease," in *High throughput protein expression and purification: methods and protocols* (Springer, 2009) pp. 297–307.

[8]S. Lövestam, J. L. Wagstaff, T. Katsinelos, J. Shi, S. M. Freund, M. Goedert, and S. H. Scheres, "Twelve phosphomimetic mutations induce the assembly of recombinant full-length human tau into paired helical filaments," eLife (2025).

[9]K. Kitoka, A. Lends, G. Kucinskas, A. L. Bula, L. Krasauskas, V. Smirnovas, M. Zilkova, B. Kovacech, R. Skrabana, J. Hritz, *et al.*, "dGae (297–391) tau fragment promotes formation of chronic traumatic encephalopathy-like tau filaments," Angew. Chem. Int. Ed. **63**, e202407821 (2024).

[10]W. P. Walters and M. A. Murcko, "Prediction of 'drug-likeness'," Adv. Drug Deliv. Rev. **54**, 255–271 (2002).

[11]O. V. Oksiuta and Y. I. Filatov, "Assessment of the commercially available chemical space for using in the 19F NMR FAXS method: a Enamine ltd. case," J. Org. Pharm. Chem. **21**, 21–28 (2023).

[12]Y. Pan, "The dark side of fluorine," (2019).

[13]G. Landrum, "Rdkit documentation," Available from: https://www.rdkit.org/docs/ (2013).

[14]T. Sander, J. Freyss, M. Von Korff, and C. Rufener, "DataWarrior: an open-source program for chemistry aware data visualization and analysis," J. Chem. Inf. Model. **55**, 460–473 (2015).

[15]F. Neese, "Software update: The ORCA program system—version 5.0," Wiley Interdisciplinary Reviews: Computational Molecular Science **12**, e1606 (2022).

[16]A. Shaka, P. B. Barker, and R. Freeman, "Computer-optimized decoupling scheme for wideband applications and low-level operation," J. Magn. Reson. **64**, 547–552 (1985).

[17]A. Lingel, A. Vulpetti, T. Reinsperger, A. Proudfoot, R. Denay, A. Frommlet, C. Henry, U. Hommel, A. D. Gossert, B. Luy, and A. O. Frank, "Comprehensive and high-throughput exploration of chemical space using broadband 19F NMR-based screening," Angew. Chem. Int. Ed. **59**, 14809–14817 (2020).

[18]J. D. van Beek, "matNMR: A flexible toolbox for processing, analyzing and visualizing magnetic resonance data in Matlab®," J. Magn. Reson. **187**, 19–26 (2007).

[19]R. Cain, R. Salimraj, A. S. Punekar, D. Bellini, C. W. Fishwick, L. Czaplewski, D. J. Scott, G. Harris, C. G. Dowson, A. J. Lloyd, *et al.*, "Structure-guided enhancement of selectivity of chemical probe inhibitors targeting bacterial seryl-tRNA synthetase," J. Med. Chem. **62**, 9703–9717 (2019).

[20]D. Liebschner, P. V. Afonine, M. L. Baker, G. Bunkóczi, V. B. Chen, T. I. Croll, B. Hintze, L.-W. Hung, S. Jain, A. J. McCoy, *et al.*, "Macromolecular structure determination using X-rays, neutrons and electrons: recent developments in Phenix," Biol. Crystallogr. **75**, 861–877 (2019).

[21]P. V. Afonine, R. W. Grosse-Kunstleve, N. Echols, J. J. Headd, N. W. Moriarty, M. Mustyakimov, T. C. Terwilliger, A. Urzhumtsev, P. H. Zwart, and P. D. Adams, "Towards automated crystallographic structure refinement with phenix.refine," Biol. Crystallogr. **68**, 352–367 (2012).

[22]P. Emsley, B. Lohkamp, W. G. Scott, and K. Cowtan, "Features and development of coot," Biol. Crystallogr. **66**, 486–501 (2010).

[23]P. Chakraborty, G. Rivière, S. Liu, A. I. de Opakua, R. Dervişoğlu, A. Hebestreit, L. B. Andreas, I. M. Vorberg, and M. Zweckstetter, "Co-factor-free aggregation of tau into seeding-competent RNA-sequestering amyloid fibrils," Nat. Commun. **12**, 4231 (2021).

[24]C. Bengs and M. H. Levitt, "Spindynamica: Symbolic and numerical magnetic resonance in a mathematica environment," Magn. Reson. Chem. **56**, 374–414 (2018).
